# The role of business in addressing the long-term implications of the current food crisis

**DOI:** 10.1186/1744-8603-4-12

**Published:** 2008-12-05

**Authors:** Derek Yach

**Affiliations:** 1 Vice President, Global Health Policy, PepsiCo, Inc. Purchase, NY, USA

## Abstract

Before the onset of the current food crisis, the evidence of a severely neglected nutrition crisis was starting to receive attention. Increased food prices are having severe impacts on the nutritional status of populations. Our current food system has evolved over decades in a largely unplanned manner and without consideration for the complexity and implications of linkages between health, nutrition, agricultural, economic, trade and security issues. The underlying causes for the nutrition crisis include the above, as well as decades of neglect with regard to nutrition, and agricultural science (especially in emerging markets); a failure of governance with respect to the major players involved in nutrition, a weak response by government donors and Foundations to invest in basic nutrition (in contrast to growing support for humanitarian aspects of food aid), and a reluctance to develop private-public partnerships. The emergence of new business models that tackle social problems while remaining profitable offers promise that the long term nutrition needs of people can be met. Businesses can have greater impact acting collectively than individually. Food, retail, food service, chemical and pharmaceutical companies have expertise, distribution systems and customers insights, if well harnessed, could leapfrog progress in addressing the food and nutrition crises. While business can do lots more, its combined impact will be minimal if a range of essential government actions and policies are not addressed. Governments need to create innovative and complementary opportunities that include incentives for businesses including: setting clear nutritional guidelines for fortification and for ready-to eat products; offering agreements to endorse approved products and support their distribution to clinics and schools; eliminating duties on imported vitamins and other micronutrients; and providing tax and other incentives for industry to invest with donors in essential nutrition and agricultural research. Currently governments in developed countries provide a wide range of incentives to the pharmaceutical industry to develop medicated solutions to nutritional problems. We need equivalent effort to be given to the development of more sustainable agricultural and food based solutions. We now face a truly global set of interlinked crises related to food that affect all people. The same degree of urgency and high level leadership and partnership seen during the Second World War is required on a global basis. This time it will need to simultaneously address agricultural, environmental and health considerations with the aim being the attainment of optimal nutrition for all within a framework of sustainable development.

## Introduction

At a recent industry review of the food crisis, Hettie Schonfeldt from the University of Pretoria provided powerful data on the direct costs and consequences of increased food prices in rural and peri-urban areas of South Africa [1]. For many families, the impact will be deadly, pushing the vulnerable into desperation. This picture is playing out worldwide in developed and developing countries. The food price crises are also nutrition crises. Yet the links between food and nutrition or between agriculture and health are often ignored as each sector goes about its work within its silos. This message was driven home superbly in Paul Roberts' recent book *The End of Food *[2]. Potentially depressing, Robert's book stimulated me to consider what we might do to build a healthier food system.

## Worsening nutrition, health and development crisis

Before the onset of the current food crisis, the evidence of a severely neglected nutrition crisis was starting to receive attention. There are globally 1 billion overweight or obese people, 1 billion who are hungry and about 2 billion people who are micronutrient deficient. 3.5 million women and children under 5 years of age die each year because of factors related to undernutrition, and many more millions of adults die prematurely due to unhealthy diets [3]. The intimate links between early childhood stunting and obesity in late childhood and adolescence is creating risks for diabetes and cardiovascular disease at younger ages in developing countries than were seen in countries that underwent a smooth epidemiological transition. The reality of a growing number of "super at risk" stunted obese people requires us to develop integrated strategies that aim at achieving optimal nutrition for all. We can no longer separate our approaches to tackling under – from over-nutrition. For most emerging economies, their causes and consequences are closely intertwined.

Increased food prices are having severe impacts on the nutritional status of populations. Globally, the World Bank estimates that doubling or more of food prices over the past 2 years is pushing 100 million people into poverty [4].

Families respond to increased prices by eating a smaller variety of foods, often of lower nutritional quality, reducing portion sizes, eating wild foods and seed stock (in rural areas), seeking credit or borrowing, begging and in time, starving. These responses compromise already extremely vulnerable populations – especially those where stunting and micronutrient deficiencies are common. Permanent declines in physical and mental growth leading to economic and broad societal impacts will usually follow.

Further, rising food prices have already led to civil strife in countries on all continents, and to growing distrust of food companies in some countries due to their perceived inaction. We have seen the television coverage over the last few months from Haiti, Bangladesh, Egypt and Mozambique [5]. And more recently we have seen some governments revert to 1970s style self-sufficiency policies by banning exports of food. For examples countries such as Vietnam, Argentina, Indonesia, Brazil, India and Egypt have adopted various export restrictions on crops such as rice and wheat [5]. Such limits have played an important role, with additional factors, in causing the prices of soy beans, wheat and corn to increase over the past year.

The causes for increased food prices have been well described by the World Bank, many academics and NGOs. Lester Brown, writing a decade ago for the Worldwatch Institute predicted almost exactly what is unfolding today [6]. Causes of the food price increases include rising oil prices, surging demand for grain (to largely produce meat) especially in China, and greater use of biofuel.

Our current food system has evolved over decades in a largely unplanned manner and without consideration for the complexity and implications of linkages between health, nutrition, agricultural, economic, trade and security issues. Many decisions taken in an uncoordinated manner, by governments, industry, academics and farmers over decades have led us to where we are today. It is easy to blame in retrospect. It's far tougher to make the bold choices needed to avoid a repeat of the past.

The underlying causes for the nutrition crisis include those highlighted above but include several nutrition specific factors. These include decades of neglect with regard to nutritional science (especially in emerging markets); a failure of governance with respect to the major players involved in nutrition, a weak response by government donors and Foundations to invest in basic nutrition (in contrast to growing support for humanitarian aspects of food aid), and a reluctance to develop private-public partnerships.

These points have been highlighted in many major reviews and meetings this year. Most notable have been the articles in a Lancet series of January 19^th ^2008 that pulled together some the most thoughtful leaders in the worlds of nutrition and public health [7]. Their view is that nutrition science has not received the support it warrants. Work that PepsiCo and Liverpool University are undertaking concludes that a tiny fraction of total nutrition science output comes from emerging markets-and virtually none from the poorest countries of the world. Well over 80 percent of all scientific output in the top medical and nutrition journals address overweight and nutrition, with about 15 percent addressing micronutrients and 5 percent focus on stunting and hunger.

In contrast, there has been progress in redressing this lack of investment in science in developing countries with respect to HIV/AIDS, showing it could be improved. Ricardo Uauy, President of the International Union of Nutrition Sciences, writing in the Lancet series, places emphasis on the impact of having a failed governance system for nutrition [8] and the World Development Report of 2008 concludes similarly with respect to agricultural science and governance [9]. Of course, there has been scant attention paid to the consequences of the schism that still divides those working on agricultural and nutrition solutions.

I mentioned there had been significant meetings that brought fresh focus to the issues of nutrition. The Pacific Health Summit is one that took part in June 2008 [10]. This meeting did start to bridge some of the divides and silos I referred to earlier.

## Emerging engagement with business

Let me now turn to a subject I am learning about daily. My transition from decades in the public sector to the private sector has opened my eyes to opportunities I could not have imagined. Ideological differences between the public and private sectors that have hampered them talking to each other, are slowly giving way to the development of creative ways of interacting.

The emergence of new business models that tackle social problems while remaining profitable offers promise that the long term nutrition needs of people can be met. Muhammad Yunus (Grameen Bank and 2007 Nobel Prize Winner) recently called for the development of "social business entrepreneurs" and backed this call by working with DANONE to develop new ways of addressing the nutrition needs of poor families in Bangladesh . In January 2008, Bill Gates (Microsoft) urged that a new form of "creative capitalism" was needed [11]. PepsiCo CEO, Indra Nooyi, defines a "good business" as one that addresses financial performance while addressing health and environmental needs. She calls this performance with purpose [12].

In May 2008, Gordon Brown, with UNDP, launched an initiative aimed at drawing on business's core capabilities to contribute to the attainment of the MDGs [13]. Several food companies responded to this call to action, and have committed to use their distribution systems to get food aid to remote areas; to develop new nutritious and affordable food products for the poorest communities; to lever their agricultural research to develop plans with higher yields; and to invest in nutrition science of benefit to the public and private sectors.

## Desired actions by business to address the long-term food and nutrition crises

Businesses can have greater impact acting collectively than individually. In May 2008 CEOs of 8 major food companies pledged in a letter to the WHO Director General to develop and market fortified nutritious products to the poorest communities (personal correspondence, 2008). This is in addition to broader commitments that CEOs made to support WHO implement the action plan of the Global Strategy on Diet and Physical Activity [14]. The companies are gearing up to develop specific steps that will demonstrate their on the ground progress. The Global Alliance for Improved Nutrition (GAIN) has brought together food, retail and pharmaceutical companies to tackle micronutrient deficiencies in innovative and exciting ways. GAIN received its initial funding from the Bill and Melinda Gates Foundations and has pioneered ways of building country specific private public partnerships [15]. Food, retail, food service, chemical and pharmaceutical companies have expertise, distribution systems and customers insights that if well harnessed, could leapfrog progress in addressing the food and nutrition crises.

Business could increasingly address the entire range of the agricultural investment climate, including access to micro-credit for small farmers, research on better seeds, training, provision of water saving irrigation systems and long term purchase guarantees. PepsiCo's work with potato farmers in Peru (where it supports the local potato industry), China and South Africa; citrus farmers in Indian Punjab; corn farmers in rural Mexico and oats farmers worldwide include these dimensions. Business could also support local sourcing and use of indigenous foods. We know that many local and underused nutrition solutions exist in the Amazon or within Ayurvedic texts, or in the menu of options used by traditional healers of South Africa. Business has the ability to bring these to scale and do so in an ethically and environmentally sound manner.

In an environment of soaring prices, businesses need to be hyper-efficient, and reduce waste along their supply chains and reduce fuel costs by bringing production closer to consumers. This is a particular problem in Africa where up to 40 percent of fresh produce is lost through poor supply chain management. Further, the potential of using nutritious components of current waste streams for affordable nutrition is being explored.

Retail chains can work with governments and food companies to develop a balanced food basket of local staples priced to be affordable to the poor. A proposal to do this is being developed in South Africa. This, combined with a food stamp program (based on the 1965 USA version and its recent updates) could ensure that food quality is not compromised as food prices increase.

All the evidence suggests that Margaret Chan, the Director General of WHO is correct when she stressed recently stated that:

"food choices are highly sensitive to price. The first items to drop out of the diet are usually the healthy foods...fatty processed foods or low-energy nutrient staples are often the cheapest way to fill hungry stomachs" [16]

What can we collectively to develop a business model that works to reverse this? We are devoting our time to thinking this through and would value all of your insights!

There is broad consensus about the need for nutrition interventions to give priority to young women and children under 2 or 3 years of age if the long-term effects of stunting on growth and intellectual development are to be prevented. Business could, and in some cases is, supporting this through the development and marketing of products for women and children that address key nutrient needs. Business, through joined up efforts with governments, could support truly effective social marketing campaigns for breast feeding. Recall that breastfeeding is the most cost-effective nutritional intervention we have and is the worst marketed intervention.

Business could support programs to fortify staples and developing a wider range of ready-to-eat therapeutic foods. The June 2008 High-Level Task Force of the United Nations on the Global Food Crisis that met in Rome included these elements in their Comprehensive Framework for Action but failed then to consider the role of multinationals as possible partners in their implementation [17]. No budgets or priorities were discussed. Rather a long and complex wish list was produced for UN agencies, most of whom are severely understaffed and underfunded, to implement!

Following the June meeting, the UN Global Compact office has completed a report highlighting the need for certain private sector actions [18]. These include a need to openly address impediments to food company involvement in addressing the complimentary food needs of children worldwide to a far greater extent. This could be done provided NGOs and governments were able to move beyond fears that food company engagement in this area would undermine the WHO International Code of Marketing Breastmilk Substitutes.

The demand for meat is a response to a lack of protein in the diet of emerging market populations. Global meat consumption is about 100 g/person/day with there being a 10 fold variation between high and low consumption countries [19]. If continued demand for protein is met with meat, the consequences for the environment, human health and vulnerable populations will be dire. Business and academia need to lead through their R&D and marketing in developing ways of stimulating increased consumption of less energy/grain intensive protein sources from plants, fish and in vitro meat cultures. As Ricardo Uauy and his colleagues stressed in the recent Lancet review, a goal of achieving a more equitably distributed global consumption of 90 g/person/day by the 2030s is possible if work started in earnest today [19]. At that level, populations' needs for animal protein and iron would be easily met.

Business has an important advocacy role to play on issues that affect agricultural productivity. This includes calling for an equitable outcome to the Doha trade round and an end to European and US farm subsidies. Business also needs to be part of the dialogue at country-level about how to create an enabling environment for investing in agriculture and local food production.

While business can do lots more, its combined impact will be minimal if a range of essential government actions and policies are not addressed.

### Governments need to create innovative and complementary opportunities that include incentives for businesses including

setting clear nutritional guidelines for fortification and for ready-to eat products; offering agreements to endorse approved products and support their distribution to clinics and schools; eliminating duties on imported vitamins and other micronutrients; and providing tax and other incentives for industry to invest with donors in essential nutrition and agricultural research. Currently governments in developed countries provide a wide range of incentives to the pharmaceutical industry to develop medicated solutions to nutritional problems. We need equivalent effort to be given to the development of more sustainable agricultural and food based solutions.

It is notable that while total government support for international health issues has increased over the last decade, almost all of the increase has been driven by HIV/AIDS investments. Basic nutrition support has actually declined just as the demand has increased.

The World Development Report of 2008 highlighted the centrality of R&D investments for agriculture [9]. Their arguments also apply to nutrition science and to research of the boundaries of agriculture, nutrition and the environment. Failure to do so will keep us digging deeper into our silos.

## Leadership and partnership

When I was at WHO working on the Global Strategy on Diet and Physical Activity, we proposed to the then heads of nutrition and agriculture at FAO that we undertake a joint piece of long-term work that aimed to consider what the agricultural supply should look like in a world of the 2030s assuming people consumed according to our ideal nutrition guidelines. We felt that the exercise itself would unify the visions and goals of agriculture and nutrition; the worlds of over nutrition and under nutrition; and the agricultural worlds of cash crop promoters and food self-sufficiency promoters. We failed to get the work done for many reasons.

Now, it seems as critical as then. But it should involve far more than WHO and FAO. It needs to take account of the reality that while our UN governance system is still based upon a 1946 perspective of the major players in the world, real action, investments and policies are driven by a much wider array of players-including Foundations, corporations and NGOs.

The recent G 8 statement on food security, strongly supported by the French Government, included a call for the creation of a global network of high-level experts on food and agriculture to provide science-based analysis, and highlight needs and future risks [20]. The linkages between agriculture, nutrition the environment were not mentioned in the communiques; and the spirit of the announcements that have been made public suggest that we might well see more of the same.

I had the opportunity to participate in a meeting in September, 2008 where the US Secretary of Defense was present. I reminded him that the poor nutritional status of soldiers early in World War II led President Franklin Roosevelt to host the National Nutrition Conference for Defense in 1941. It called for development of a new kind of flour capable of improving the physical and mental stamina of soldiers-private millers responded. Secretary Gates reminded us that we face dual threats of under- and over-nutrition. Both have implications for security. Overweight and obesity constitute a current threat to the recruitment of soldiers with 23 percent of potential recruits not meeting health and nutrition standards; and stunting and hunger constitute a threat to stability and security in many countries.

We now face a truly global set of interlinked crises related to food that affect all people. The same degree of urgency and high level leadership and partnership seen during the Second World War is required on a global basis. This time it will need to simultaneously address agricultural, environmental and health considerations with the aim being the attainment of optimal nutrition for all within a framework of sustainable development.

This paper is based on a speech given at the Forum on Global Food Systems. Sept 16^th ^2008. Earth Institute, Columbia University, New York USA.

## Competing interests

The author declares that he is currently employed by PepsiCo, Inc.
